# Potential Molecular Biomarkers of Preeclampsia—A Pilot Study

**DOI:** 10.3390/ijms26136149

**Published:** 2025-06-26

**Authors:** Anna Romała, Eliza Matuszewska-Mach, Wiesław Markwitz, Maciej Brązert, Paulina Borysewicz, Dagmara Pietkiewicz, Jan Matysiak, Krzysztof Drews, Agata Szpera

**Affiliations:** 1 Department of Perinatology, Poznan University of Medical Sciences, Polna 33 Street, 60-535 Poznan, Poland; wmarkwitz@ump.edu.pl (W.M.); agata.szpera@gmail.com (A.S.); 2 Department of Inorganic and Analytical Chemistry, Poznan University of Medical Sciences, Rokietnicka 3 Street, 60-806 Poznan, Poland; eliza.matuszewska@ump.edu.pl (E.M.-M.); dagmarapietkiewicz3@gmail.com (D.P.); jmatysiak@ump.edu.pl (J.M.); 3 Department of Diagnostics and Treatment of Fertility, Poznan University of Medical Sciences, Polna 33 Street, 60-535 Poznan, Poland; maciejbrazert@gmail.com; 4 The Students Scientific Society, Poznan University of Medical Sciences, Rokietnicka 5 Street, 60-806 Poznan, Poland; borysewiczpaulina@gmail.com; 5 Faculty of Medicine, Poznan Medical University, Bułgarska 55 Street, 60-320 Poznan, Poland; kdrews@gpsk.am.poznan.pl

**Keywords:** calbindin 1, clusterin, GSTP1, KIM-1, MCP-1, IL-18, preeclampsia, biomarkers

## Abstract

Preeclampsia, one of the leading causes of maternal and fetal morbidity and mortality, affects approximately 3–5% of pregnancies worldwide. However, its etiology remains poorly understood. The aim of this study was to identify molecular markers of preeclampsia. Protein concentrations in blood and urine were determined using the Bio-Plex Kidney Toxicity 1 assay Bio-Rad, Hercules, CA, USA followed by magnetic separation and flow cytometry. This study included 51 patients with preeclampsia and 25 healthy pregnant women. The results revealed that five out of the six serum biomarkers of kidney injury were elevated in the preeclampsia group compared to the control group (calbindin 1, clusterin, glutathione transferase pi (GSTP1), monocyte chemotactic protein 1 (MCP-1), and kidney injury molecule type 1 (KIM-1)). Additionally, the serum concentrations of calbindin 1, clusterin, GSTP1, and KIM-1 were significantly higher in both early-onset and late-onset preeclampsia compared to the control group. The analysis of urinary proteins showed that only the KIM-1 concentration was elevated in late-onset preeclampsia compared to the control group. These findings suggest that the calbindin 1, clusterin, GSTP1, KIM-1, and MCP-1 concentrations in maternal plasma could serve as potential biomarkers for monitoring kidney injury in preeclamptic women. This study provides a foundation for future research to explore novel biomarkers of preeclampsia and renal injury in pregnant women.

## 1. Introduction

Preeclampsia (PE) is an obstetric disorder which is defined as hypertension along with one or more of the following: proteinuria, maternal organ dysfunctions (of renal, liver, neurological, or hematological nature), uteroplacental dysfunction such as fetal growth restriction, stillbirth, and an imbalance of angiogenic markers [1,2,3]. Morbidity as well as PE-associated mortality affects 5% to 7% of all pregnant women and is the cause of over 70,000 maternal and 500,000 fetal deaths worldwide each year [4].

Two types of PE can be distinguished according to gestational age at the time of diagnosis. The first one is early-onset preeclampsia (EO-PE), which usually occurs at <34 weeks of pregnancy and is characterized by significant fetal morbidities. The pathophysiology is believed to involve impaired trophoblast invasion of the maternal spiral arterioles, leading to persistent high-resistance maternal vasculature. This is consistent with the high frequency of placental underperfusion reported in this disorder [2]. In EO-PE, there is a higher risk of the HELLP (Hemolysis, Elevated Liver enzymes, Low Platelet count) syndrome, an abnormal uterine artery Doppler waveform, atherosis, placental lesions, small-for-gestational-age newborns, and fetal growth restriction [4,5,6,7,8]. Even 80–90% of women with EO-PE have abnormal maternal plasma soluble Fms-like Tyrosine Kinase-1-to-placental growth factor (sFlt-1:PlGF) or placental growth factor-to-soluble Endoglin (PlGF:sEng) ratios; this imbalance affects only 40–50% of patients with late-onset preeclampsia [9,10].

The second type—late-onset preeclampsia (LO-PE)—is thought to be more of a maternal constitutional disorder due to underlying maternal microvascular disorders such as hypertension or a genetic predisposition. Here, poor trophoblast invasion is not as important as in the early-onset type. LO-PE is significantly more common, and while it often has a mild course, it can be associated with significant clinical morbidities [2]. Nowadays, multiple lines of evidence indicate that EO-PE and LO-PE are two different conditions.

The current diagnostic criteria for PE include the guidelines set by the International Society for the Study of Hypertension in Pregnancy (ISSHP). Pregnant women with hypertension ought to be tested in terms of their levels of hemoglobin, platelet count, serum creatinine, liver enzymes, and serum uric acid [3]. Abnormal values could indicate any kind of maternal organ dysfunction. In terms of hypertension, both the diastolic and systolic values are of equal importance in the diagnosis [11]; a value of 140/90 mm/Hg is the limit, and any number over that is related to a rise in perinatal complications [12]. In preeclamptic women, there is an overproduction of sFlt-1 and decreased levels of PlGF [13]. These two biomarkers should not be evaluated alone but rather as a ratio, as a high ratio of sFlt-1 to PlGF is actually related to a higher risk of developing PE [14]. The most recent data suggest that enhancing the definition of PE to include the sFlt-1:PlGF ratio can better identify women and babies at risk of adverse outcomes [15].

Nonetheless, currently, scientists are trying to find more sensitive biomarkers in order to diagnose PE. Some studies have used metabolomic techniques like Liquid Chromatography–Mass Spectrometry (LC-MS), Ultra-Performance Liquid Chromatography (UPLC), and Magnetic Resonance (MR) [16,17]. According to the available literature, the trend is to look for biomarkers in features of the metabolome. Modern research techniques are enabling the search for new, early, and sensitive markers of kidney damage in PE. Disease processes lead to changes in the expression of genes encoding proteins that can serve as potential biomarkers. Using transcriptomic, proteomic, and metabolomic techniques, a number of characteristic proteins in the urine and serum have been identified in the course of kidney damage. These include calbindin 1, clusterin, glutathione transferase pi (GSTP1), interleukin 18 (IL-18), kidney injury molecule type 1 (KIM-1), and monocyte chemotactic protein 1 (MCP-1) [18,19,20,21,22,23,24,25,26,27,28,29,30,31,32,33].

In preeclamptic women, the imbalance between pro- and anti-angiogenic factors is responsible for kidney injury. On the other hand, glomerulonephritis compromises kidney function, makes kidneys less responsive to the physiological changes accompanying pregnancy, and causes vascular inflammation in pregnant women—a risk factor for PE development [34]. Despite a demonstrated bidirectional correlation between glomerulonephritis and PE, the data are limited, and further studies are needed.

Therefore, this paper focuses on finding the potential molecular markers of renal injury, which is one of the characteristics of PE, the main one being hypertension. Protein concentrations in the blood and urine were analyzed using the Bio-Plex Pro™ RBM Human Kidney Toxicity Panel 1 Bio-Rad, Hercules, CA, USA, as this multiplex assay targets biomarkers specifically associated with renal injury. Bio-Plex Multiplex immunoassays Bio-Rad, Hercules, CA, USA use fluorescently dyed magnetic beads for the quantification of a biologically relevant target, based on the Luminex/xMAP technology [35]. This technology may provide a more efficient and rapid approach to help determine if a patient has PE.

## 2. Results

Serum and urine samples were assessed using the Bio-Plex Pro RBM Human Kidney Toxicity Panel 1 immunoassay Bio-Rad, Hercules, CA, USA. The panel contains six proteins (calbindin 1, clusterin, GSTP1, IL-18, KIM-1, and MCP-1). Table 1 shows the descriptive characteristics of the obtained concentrations in the serum and urine of all 76 women studied. Interestingly, the concentrations of five of the analyzed proteins were higher in the serum than in the urine; only for GSTP1 were the concentrations higher in the urine.

The obtained concentrations in the test and control groups were then analyzed. Since the distribution of the values of the evaluated proteins did not show conformity to a normal distribution assessed by the Shapiro–Wilk test, the analysis of statistical differences between the groups was carried out using non-parametric tests. The results of the concentrations obtained for the groups of women with PE and the control group are shown in Table 2.

All serum concentrations of biomarkers of kidney damage determined in the PE group were higher in the PE group compared to in the control group, and statistically significant differences were obtained for five of them. The median serum concentration of calbindin in patients with PE was 925.12 ng/mL, while in the control group, it was 838.47 ng/mL (*p* = 0.0024). There was a statistically significant difference in the clusterin levels between the PE group and the control group (*p* = 0.0018). The medians were 124,693.50 ng/mL (interquartile range (IQR): 97,071.73–154,823.30) in the study group and 96,323.58 ng/mL (IQR: 69,449.51–116,498.70) in the control group. The GSTP1 protein concentration was significantly higher in the group of patients with PE (*p* = 0.0001); it was 19.2 ng/mL. In the group of healthy female patients, it was equal to 13.08 ng/mL. There was no significant difference in the mean concentration of IL-18 between the groups (*p* = 0.1942). In the serum of patients with PE, it was equal to 2.61 ng/mL, and in the control group, it was equal to 2.41 ng/mL. The concentration of the KIM-1 protein in the serum of pregnant women with PE was 3.25 ng/mL, while in the group of healthy pregnant women, it was equal to 3.03 ng/mL. There was a statistically significant difference between the groups (*p* = 0.0042). For the MCP-1 protein, the median in the PE group was 0.99 ng/mL, while in the control group, it was equal to 0.84 ng/mL. The difference between the groups was statistically significant (*p* = 0.0228).

There were no statistically significant differences in the urinary protein concentrations of the patients in the PE and control groups (Table 2).

**Figure 1:**
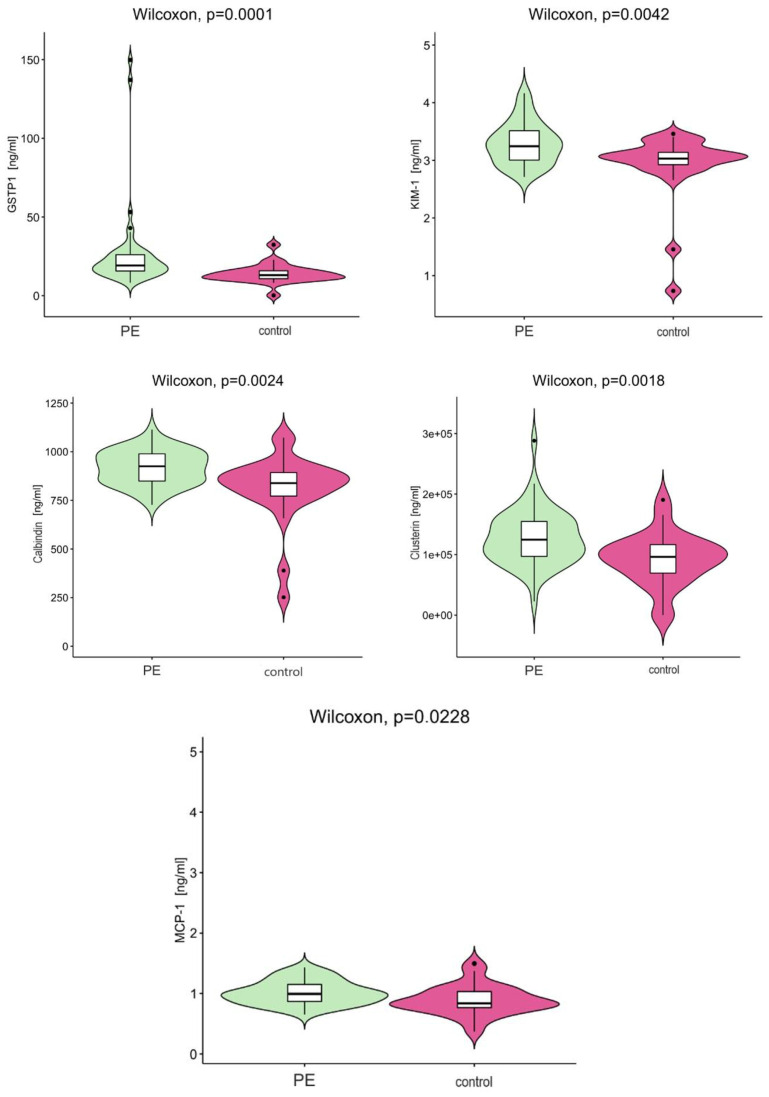
Statistically significant differences in serum protein concentrations for PE and control groups.

The statistically significant differences between the PE and control groups discussed are shown schematically in Figure 1.

Table 3 shows the results of statistical tests comparing the serum and urine concentrations of the studied proteins of patients in the EO-PE, LO-PE, and control groups. The serum showed statistically significant differences between the EO-PE, LO-PE, and control groups in the protein levels of calbindin 1 (*p* = 0.0095), clusterin (*p* = 0.0071), GSTP1 (*p* = 0.0003), and KIM-1 (*p* = 0.0235). Post hoc analysis showed that for these four proteins, the concentrations were statistically different between both the EO-PE and LO-PE groups and the control group. No statistically significant differences were observed between the groups of pregnant women with EO-PE and LO-PE.

**Figure 2:**
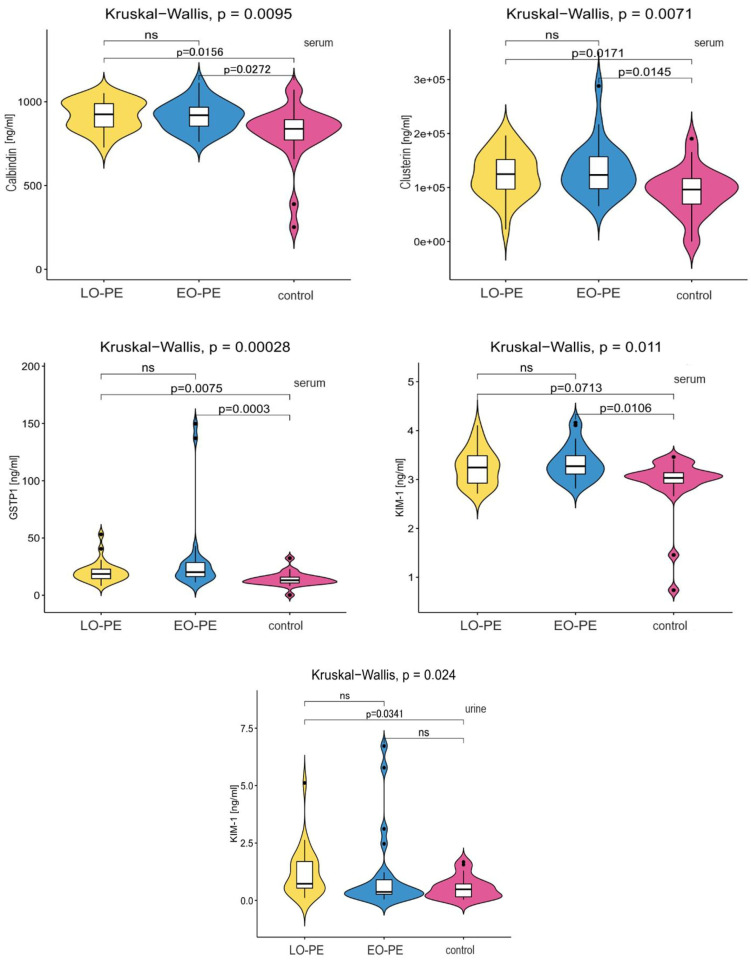
Statistically significant differences in protein concentrations in the EO-PE, LO-PE, and control groups.

The urine of pregnant women showed no significant difference in the levels of the following proteins: calbindin 1 (*p* = 0.5449), clusterin (*p* = 0.2978), GSTP1 (*p* = 0.5409), IL-18 (*p* = 0.1929), and MCP-1 (*p* = 0.3325). Between the study groups, there was a statistically significant difference only in the concentration of the KIM-1 protein (*p* = 0.0235). Its concentration did not differ between the EO-PE and control groups (p* = 0.6349) or between the LO-PE and EO-PE groups (p* = 0.0730). However, there was a statistically significant difference in the urinary KIM-1 concentration between the LO-PE group and the control group (p* = 0.0341). Figure 2 shows the statistically significant differences in the EO-PE, LO-PE, and control groups.

### 2.1. Correlations of Biomarkers of Kidney Damage

The present study also estimated the correlations occurring between the studied proteins in the blood serum and urine of the studied women and the proteins and clinical data of mothers and newborns. To analyze the existing correlations, the non-parametric Spearman test with Holm’s correction for multiple comparisons was used.

In the evaluation of the correlations between the biomarkers of kidney damage in the entire group of 76 women studied, there were no statistically significant correlations between the concentrations of proteins obtained from the serum and proteins determined in the urine.

Some of the correlations between serum proteins were statistically significant. The strongest correlation was noted for calbindin 1 and clusterin (rho = 0.84; *p* < 0.001), but a *p*-value < 0.001 was obtained for calbindin and the other proteins: GSTP1, IL-18, KIM-1, and MCP-1. Clusterin did not correlate statistically significantly only with IL-18 (rho = 0.36; *p* = 0.052). For GSTP1, rho = 0.42 was obtained with IL-18 (*p* = 0.006), rho = 0.43 with KIM-1 (*p* = 0.004), and rho = 0.33 with MCP-1 (*p* = 0.123). Strong correlations were shown between IL-18 and KIM-1 (rho = 0.61; *p* < 0.001) and MCP-1 (rho = 0.64; *p* < 0.001).

**Figure 3:**
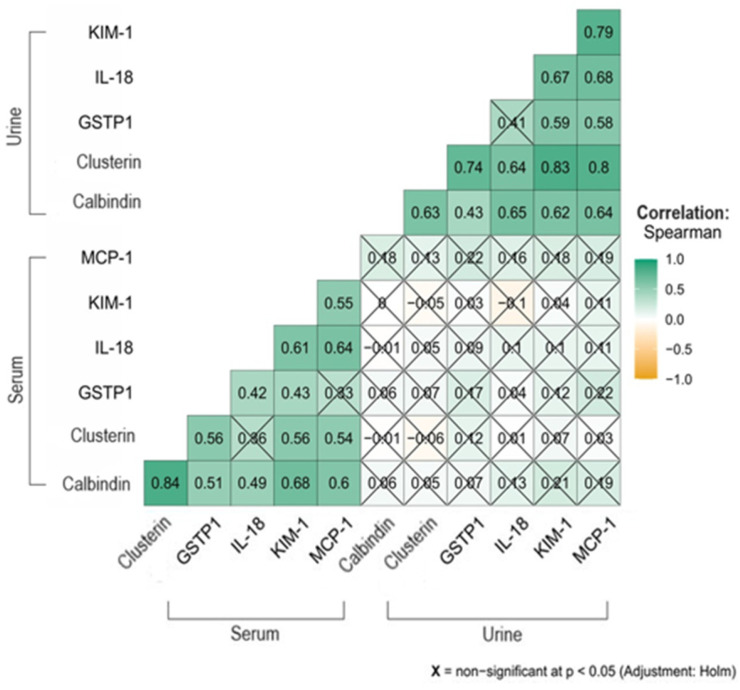
The correlations occurring between the studied proteins quantified in the patients’ serum and urine.

There were also very strong correlations between the proteins determined in the patients’ urine, most *p* < 0.001. The weakest were noted for calbindin 1 and GSTP1 (rho = 0.43; *p* = 0.037) and for GSTP1 and IL-18 (rho = 0.41; *p* = 0.095). The correlations between proteins for the entire study group discussed are shown schematically in Figure 3.

In evaluating the correlations between serum proteins and clinical data, a statistically significant negative correlation was found for clusterin and KIM-1 with gestational age at delivery (rho = −0.37, *p* = 0.019, and rho = −0.34, *p* = 0.045, respectively). Positive correlations were found between IL-18 and BMI (Body Mass Index) before pregnancy (rho = 0.37; *p* = 0.024), MCP-1 and BMI at the end of pregnancy (rho = 0.39; *p* = 0.013), calbindin 1 and systolic blood pressure (rho = 0.35; *p* = 0.046), and KIM-1 and systolic (rho = 0.9; *p* = 0.010) and diastolic blood pressure (rho = 0.36; *p* = 0.024). There were no statistically significant correlations between the analyzed proteins from the patients’ urine and the clinical data.

After dividing the whole group into patients with PE and healthy pregnant women for serum proteins, statistically significant differences were observed in both groups for the correlations of clusterin with calbindin, KIM-1 with IL-18 and MCP-1, and IL-18 with MCP-1. In the PE group, statistically significant correlations were found between calbindin and IL-18 (rho = 0.63; *p* < 0.001), KIM-1 (rho = 0.72; *p* < 0.001), and MCP-1 (rho = 0.63; *p* < 0.001) and between clusterin and GSTP1 (rho = 0.49; *p* = 0.013), KIM-1 (rho = 0.58; *p* < 0.001), and MCP-1 (rho = 0.58; *p* < 0.001), which were not present in the control group. For urine proteins, numerous statistically significant differences were observed in both groups. For both groups, there was no correlation between GSTP1 and calbindin and IL-18. In the study group with PE, statistically significant correlations were observed between IL-18 and KIM-1 (rho = 0.68; *p* < 0.001), MCP-1 (rho = 0.67; *p* < 0.001), and clusterin (rho = 0.62; *p* = 0.002) and between calbindin and MCP-1 (rho = 0.67; *p* < 0.001); these differences were not found in the control group (Table 4).

Table 4 shows the results of the correlation in the serum and urine proteins of patients with PE and the controls. Table 5 presents the correlations between different marker proteins in the subgroups of EO-PE and LO-PE. The correlations are measured using the Spearman’s rank correlation coefficient (rho), and the *p*-values indicate the statistical significance of these correlations. Calbindin and clusterin show strong positive correlations in both the PE and control groups, as well as in the EO-PE and LO-PE subgroups. IL-18, KIM-1, and MCP-1 are also correlated, especially in the PE and LO-PE groups, suggesting their role in PE. These findings could help in understanding the biological pathways involved in PE and potentially in identifying biomarkers for diagnosis or prognosis.

In analyzing the correlations of serum and urine biomarkers with clinical data such as BMI before pregnancy, BMI at the end of pregnancy, the duration of the pregnancy, newborn weight, the maximum systolic pressure, and the maximum diastolic pressure for the groups in question, no statistically significant differences were observed.

### 2.2. Predictive Value of Serum Concentrations of Analyzed Proteins for the Occurrence of Preeclampsia

ROC curves were plotted for the serum and urine concentrations of proteins that are biomarkers of kidney damage, the areas under the ROC curve (AUCs) were calculated, and cut-off points for the occurrence of PE were determined. Detailed results of the predictive value assessment are shown in Table 6.

## 3. Discussion

As PE is a serious obstetric disorder that may eventually lead to renal failure, finding biomarkers for it is of the utmost importance to treat women burdened with this disorder. The Bio-Plex Pro RBM Human Kidney Toxicity Panel 1 Bio-Rad, Hercules, CA, USA, enables the quantitative analysis of the following six proteins: calbindin, clusterin, GSTP1, KIM-1, IL-18, and MCP-1. It shows promise as a tool which allows physicians to obtain quick results on the state of preeclamptic women’s kidneys. Our data, as well as the data obtained by other researchers, shows that certain proteins are significantly higher in preeclamptic women, thus proving the usefulness of the Bio-Plex PanelBio-Rad, Hercules, CA, USA. Though useful, more studies should be conducted with serial determinations of proteins through the Bio-Plex Pro RBM Human Kidney Toxicity Panel 1 Bio-Rad, Hercules, CA, USA in conjunction with the concentrations of proteins that are markers of angiogenesis to assess the dynamics of the development of PE.

The comparative analysis of the concentrations of the determined markers showed that there are statistically significant differences in the protein concentrations determined in the blood sera (calbindin, clusterin, GSTP 1, KIM-1, and MCP-1) and in the urine (KIM-1 but not statistically significant) between the study group (PE) and the control group. In addition, there are statistically significant differences in the protein concentrations between the EO-PE and LO-PE groups, determined in the blood serum (calbindin, clusterin, GSTP-1, KIM-1) and the urine (KIM-1).

### 3.1. Calbindin

Calbindin, classified as the protein that differentiates the control and PE groups (both LO-PE and EO-PE), is also known as calbindin-28K, an intracellular protein that has the ability to bind calcium ions. In the nephron, it is found in the distal tubule and in the proximal part of the collecting tubule [36]. It is involved in active calcium resorption. Damage to the distal part of the nephron determines the altered expression of calbindin 1 and its excretion in the urine [37]. In our study, the serum concentrations of calbindin suggest that there is a statistically significant difference between patients with EO-PE and LO-PE and healthy pregnant women. In the groups of pregnant women with PE, the concentration of this protein was significantly higher than in the control group. This study is the first to report on the serum levels of calbindin 1 in patients with PE; however, adequate data comparison is not possible as there are no other studies published concerning this matter.

On the other hand, the urinary concentration of calbindin in pregnant women with PE showed no statistically significant difference from that in the control group. There was also no statistically significant difference in the urine concentrations between the EO-PE, LO-PE, and control groups. However, in the study conducted by Eltounali et al., urine samples were used and also assessed using the Bio-Plex technology [18] in preeclamptic women that were HIV-positive and HIV-negative. The conclusion was that the calbindin levels were elevated in all women with PE regardless of being HIV-positive or HIV-negative [18]. This shows promise for calbindin as a potential PE biomarker in the future; however, more evidence and data are needed.

### 3.2. Clusterin

Clusterin is a multifunctional glycoprotein associated with apoptosis, hypoxia, oxidative stress, and mechanical tissue damage, among other processes. Its increased expression is observed in neurodegenerative diseases as well as in renal diseases [38]. Nonetheless, the reasons for the elevated levels of clusterin in the serum of patients with PE are not completely understood. It has been speculated that the overexpression of this protein in PE may result from endothelial cell damage, renal dysfunction in PE, oxidative stress, or its release from platelet alpha-granules in PE [39]. Our analysis showed a statistically significant difference in clusterin levels between the group of patients with PE and healthy pregnant women. The difference was also shown between the LO-PE and control and EO-PE and control groups. The serum clusterin concentrations of patients in the EO-PE versus LO-PE groups were similar.

Our results are consistent with studies conducted by the teams of Watanabe et al. and Oztas et al. conducted a study on a group of patients with PE and healthy pregnant women using proteomic techniques such as two-dimensional electrophoresis (2-DE) and matrix-assisted laser desorption/ionization–time-of-flight mass spectrometry (MALDI-TOF-MS). They observed a significantly higher concentration of clusterin in the group of pregnant women with PE compared to the healthy control group [39]. Equally, Oztas et al. conducted a study on a group of preeclamptic and healthy pregnant women. The researchers observed statistically significantly higher concentrations of clusterin in the serum of patients with PE compared to in healthy pregnant women [19].

In contradiction to our determinations are the results obtained by the team of Mlambo. They analyzed the serum clusterin levels in groups of patients with PE and healthy, non-hypertensive pregnancies in relation to HIV infection status. The researchers found no statistically significant difference between the groups of patients with PE and the control group regardless of HIV status, and the lowest clusterin levels were observed in the group of HIV-negative patients with PE [20]. The differences may be due to discrepancies in the gestational age of the patients studied, the burden of HIV infection in part of the population, and the ethnicity of the patients.

There were no statistically significant differences in the urinary clusterin concentrations between the group of pregnant women with PE and the control group. Concentrations in the LO-PE, EO-PE, and control subgroups were also not statistically significantly different. In contrast to this, studies by Lopez-Hernandez et al. and Odun-Ayo et al. both showed statistically significantly higher clusterin levels in women with PE [21].

### 3.3. Glutathione Transferase Pi (GSTP1)

GSTP1 is an antioxidant enzyme involved in detoxification processes, found in the distal tubule of the nephron. It has been shown to be expressed in many tissues, particularly in the bile ducts, lungs, and kidneys. Elevated levels of GSTP1 expression are found in many human cancers. Our study showed significantly higher serum GSTP1 protein levels in pregnant women with PE compared to in healthy controls. Statistically significantly higher concentrations of this protein were also obtained in the LO-PE and EO-PE subgroups with respect to the control group. The concentrations in the EO-PE and LO-PE subgroups were similar.

Knapen et al. showed statistically significantly higher plasma concentrations of GSTP1 in pregnant women with PE and HELLP syndrome compared to in a healthy control group [23].

In contrast, Parveen et al. examined the levels of the lipid peroxidation product malondialdehyde (MDA) and various free radical metabolizing enzymes in patients with PE. The study group consisted of pregnant women with PE; the control group consisted of healthy pregnant women. The researchers showed a statistically significantly higher concentration of MDA and a significantly lower concentration of GST in the plasma of patients with PE [24]. Additionally, the study by Lavanya et al. analyzed the serum GST levels in women with PE, patients who developed eclampsia, and healthy pregnant women. The researchers showed significantly higher serum GST concentrations in healthy pregnant women compared to in the group burdened with hypertensive conditions. Higher concentrations of the protein were also obtained in the group of patients with PE compared to in the group of pregnant women with eclampsia [25].

Scientific reports on the serum or plasma GST levels in pregnant women with PE are inconclusive. The results obtained by our team are analogous to those of Knapen’s research team. Elevated serum GSTP1 levels in patients with PE may be due to oxidative stress developing from ischemia and hypoxia of the placental tissue. The difference between the results presented in this paper and those obtained by Parveen et al. and Lavanya et al. may be due to the fact that these researchers determined the GST protein levels in their work without distinguishing its isoforms.

In the present study, the GSTP1 protein levels in the urine of pregnant women with PE were not significantly different from those in the healthy control group. There were also no significant differences in the urinary GSTP1 concentrations between the EO-PE, LO-PE, and control subgroups.

The results are in agreement with the analyses of Roes’s research team. Roes et al. analyzed urinary GSTP1 protein levels and found that the concentration of this protein was similar in the study groups of pregnant women with severe PE as well as those with uncomplicated pregnancies [26]. The lack of significant differences in the urinary GSTP1 concentrations in patients with PE compared to in healthy pregnant women may suggest that the metabolism of this protein in the distal tubule of the nephron does not change in the course of PE.

The aforementioned study by Odun-Ayo et al. also examined urinary GSTP1 levels and showed elevated levels of this protein in pregnant women with PE compared to in a group of normotensive pregnant women regardless of HIV infection status. However, the difference shown by the team was not statistically significant [22].

### 3.4. Interleukin 18 (IL-18)

IL-18 is a pro-inflammatory cytokine that is synthesized primarily by macrophages and monocytes. Among other things, it is believed to be responsible for regulating both innate and acquired immunity and stimulating Th1 immune responses. IL-18 in urine is a specific marker of renal injury depicting ischemic damage within the proximal tubules [18]. In the study material, the serum IL-18 levels in the group of pregnant women with PE were similar to those in the control group. There was also no significant difference between the subgroups of EO-PE and LO-PE and the control group.

Huang et al. showed elevated IL-18 concentrations in both the serum and placenta in pregnant women with PE; in the analyses by Adams et al. and Roland et al., the concentrations in the PE group were significantly lower, while Xiao showed no statistically significant difference in the PE group. A meta-analysis by Yang et al. analyzing 11 studies on IL-18 concentrations in pregnant women with PE showed no statistically significant difference between the group of patients with PE and the control group. Our analysis is in agreement with the study of Xiao’s team as well as the results of the meta-analysis of Yang et al. The variation in the results obtained may be related to the different size and composition of the groups studied, as well as the statistical methods used—some studies compared mean concentrations and others compared median concentrations. The preparation methodology of the samples studied may also have affected the release of pro-inflammatory mediators from blood cells [27,28,29,30,31].

As IL-18 is a pro-inflammatory cytokine produced in kidney cells, its increased concentration is considered a specific marker of acute renal tubular necrosis in the course of the renal ischemic process. The results obtained by most teams may indicate an increased inflammatory response in the kidney during physiological pregnancy.

In our analysis, the urinary IL-18 levels in the group of patients with PE were similar to those in the control group. There was also no significant difference between the EO-PE, LO-PE, and control subgroups.

In the previously cited study, Lopez-Hernandez et al. also analyzed the urinary IL-18 levels in pregnant women with PE. The researchers found no statistically significant difference in the urinary IL-18 concentrations between the group of patients burdened with PE and a healthy control group [21]. Eltounali et al., in the paper cited above, studied the concentration of IL-18 in the urine of patients with PE. The team found no statistically significant difference in the IL-18 concentrations between the groups analyzed [18]. Then, in the study by Xiao et al., the researchers showed a statistically significantly higher concentration of IL-18 in the urine of patients with PE [30]. Therefore, the inconclusive data suggests that measuring the concentration of IL-18 in the urine may not be a reliable biomarker for PE.

### 3.5. Kidney Injury Molecule Type 1 (KIM-1)

KIM-1 is a specific marker of proximal tubule nephron damage [40]. Proximal tubule epithelial cells increase the secretion of this protein in response to ischemia and renal tissue damage. KIM-1 influences the transformation of proximal tubule epithelial cells into phagocytes, which act like macrophages to remove apoptotic debris from locally damaged tissue [41]. The present study showed statistically significantly higher serum levels of KIM-1 protein in patients with PE compared to in healthy pregnant women. Further analysis showed significantly higher concentrations in the EO-PE and LO-PE subgroups compared to in the control group. The serum concentrations of KIM-1 in pregnant women with EO-PE and LO-PE were similar.

As there are no papers in the PubMed database analyzing the concentration of KIM-1 in the serum of pregnant women with PE, our study appears to be the first analysis of KIM-1 protein levels in the serum of pregnant women with PE and may set a trend for further research.

The available studies suggest that the concentration of the KIM-1 protein in the urine can be used as a biomarker to assess renal damage in PE. The severe form of PE is characterized by higher concentrations of this protein, which may indicate the severity of renal tissue damage in its course. From the results of the study by Lopez-Hernandez et al., it can be concluded that the change in the concentration of KIM-1 in the urine of patients with PE compared to physiologically normal pregnancies appears after 20 weeks of pregnancy [21].

When it comes to the urinary concentrations obtained in our study, the concentration of KIM-1 in the urine of patients with PE was higher than in the control group. However, the difference obtained was not statistically significant. Comparing the concentration of KIM-1 in different subgroups, its concentration was found to be highest in the urine of patients in the LO-PE group compared to in the healthy control group, and the obtained difference was statistically significant. There was no significant difference between the LO-PE and EO-PE groups as well as between the EO-PE and healthy control groups.

### 3.6. Monocyte Chemotactic Protein 1 (MCP-1)

MCP-1 plays an important role in tumor angiogenesis and the maintenance of normal pregnancy from implantation to delivery [42]. This protein is also known as a mediator of acute ischemic kidney injury. In vitro studies have shown that MCP-1 is a specific activator of tubular epithelial cells. This protein correlates with renal function scores and albuminuria [43]. MCP-1 is expressed in renal tubular epithelial cells and in white blood cells that infiltrate the renal interstitium. These cells are therefore the source of MCP-1 in the urine [44].

In our study, significantly higher MCP-1 levels were obtained in the serum of patients with PE compared to in healthy pregnant women. There were no statistically significant differences in the concentration of this protein in the serum of pregnant women in the EO-PE, LO-PE, and healthy control groups.

A similar finding was described in the study by Katabuchi et al., where women burdened with PE showed statistically significantly higher plasma levels of the MCP-1 protein compared to healthy pregnant women [32]. Likewise, Szarka et al. analyzed the serum MCP-1 levels in 60 patients with PE, 60 healthy pregnant women, and 59 healthy non-pregnant women. The researchers found statistically significantly higher serum concentrations of the tested protein in patients with PE compared to in the group of healthy pregnant women [33].

In our study, the urinary MCP-1 levels in patients with PE were similar to those in healthy pregnant women. There was also no significant difference between the EO-PE, LO-PE, and control subgroups. A similar finding was described in the aforementioned studies by Eltounali et al.—the researchers also studied the concentration of MCP-1 in the urine of pregnant women with PE. They found no statistically significant difference between the analyzed groups [18]. The work by Lopez-Hernandez et al. also analyzed the concentration of MCP-1 in the urine. The study showed no statistically significant difference in the urine of pregnant women with PE compared to the control group [21].

None of the studies conducted to date show an association between the urinary MCP-1 protein levels and the occurrence of PE. Therefore, it can be assumed that MCP-1 secretion does not correlate with renal tissue damage in PE.

### 3.7. Study Limitations

Our study has several limitations. First, the sample size was small. Second, we did not perform an assessment of the sFlt:PlGF ratio. This was because the blood and urine used in our work were collected before the publication of Verlohren’s work in which the cut-off values for the sFlt:PlGF ratio in the clinical setting of PE were given [15]. Third, we did not repeat the biomarker measurements again, which could show changes in the molecules’ levels over time depending on the severity of symptoms. Thus, trials with larger sample sizes are warranted.

## 4. Materials and Methods

### 4.1. Study Groups

This study was approved by the Bioethical Commission of Poznan University of Medical Sciences, number 1126/18. This study included 76 pregnant participants hospitalized at the Gynecological Obstetric Clinical Hospital of the Poznań University of Medical Sciences in the period between 2018 and 2021. The women were given detailed information about the aims of this study as well as the range of the study, and they all signed consent forms to consciously take part in this study.

The study group consisted of 51 patients with PE diagnosed according to the ISSHP guidelines. Table 7 contains the comparative characteristics of the PE and control patients. Out of the 51 PE patients, 24 patients were diagnosed with EO-PE and 27 with LO-PE. Table 8 contains the comparative characteristics of PE patients divided into EO-PE and LO-PE as well as healthy patients.

The control group consisted of the remaining 25 patients whose pregnancy was described as uncomplicated/healthy.

All the women were enrolled in this study based on the following criteria:

- Signing the consent form;
- Singleton pregnancy;
- Caucasian race;
- Aged over 18;
- Occurrence of PE based on the ISSHP guidelines (the criterion of inclusion into the study group).

**Table 7:** Comparative characteristics of women with PE and control women.

Clinical Data of Patients	PE Group (N = 51)	Control Group (N = 25)	*p*
Age (years), mean ± SD	29.55 ± 5.35	31.32 ± 4.11	0.1496
Pre-pregnancy weight (kg), mean ± SD	68.67 ± 13.32	63.40 ± 10.45	0.0877
Weight at the end of pregnancy (kg), mean ± SD	83.24 ± 15.78	77.04 ± 11.28	0.0838
Difference in body weight (kg), mean ± SD	14.57 ± 5.76	13.64 ± 3.25	0.3730
BMI before pregnancy (kg/m^2^), mean ± SD	25.22 ± 4.41	22.41 ± 3.32	0.0062
BMI before pregnancy, n (%)			
<18.5, underweight	2 (3.9%)	2 (8.0%)	0.0038 *
18.5–24.9, normal	25 (49.0%)	21 (84.0%)	
25.0–29.9, overweight	17 (33.3%)	1 (4.0%)	
>30.0, obese	7 (13.7%)	1 (4.0%)	
BMI at the end of pregnancy (kg/m^2^), mean ± SD	30.62 ± 5.07	27.24 ± 3.49	0.0011
BMI difference (kg/m^2^), mean ± SD	5.40 ± 2.05	4.83 ± 1.10	0.1163
Systolic blood pressure (mmHg), mean ± SD	171.70 ± 10.69	125.56 ± 7.48	<0.0001
Diastolic blood pressure (mmHg), mean ± SD	105.18 ± 7.36	80.48 ± 4.81	<0.0001
Week of pregnancy termination, mean ± SD	34 ± 3	39 ± 1	<0.0001
Days of pregnancy (gestation), mean ± SD	242 ±19	275 ± 7	<0.0001
Number of births, n (%)			
Primiparas	39 (76.5%)	15 (60.0%)	0.2238 **
Multiple births (2 to 6 pregnancies)	12 (23.5%)	10 (40.0%)	
Method of pregnancy termination, n (%)			
Cesarean section	49 (96.1%)	10 (40.0%)	<0.0001 *
Spontaneous labor	0 (0.0%)	10 (40.0%)	
Vacuum extractor	2 (3.9%)	5 (20.0%)	

* *p*—Fisher’s exact test; ** *p*—Chi-square.

**Table 8:** Comparative characteristics of women with EO-PE and LO-PE and control women.

Clinical Data of Patients	Control Group (N = 25)	EO-PE (N = 24)	LO-PE (N = 27)	*p*	*p* *
Age (years), mean ± SD	31.32 ± 4.11	30.33 ± 5.35	28.85 ± 5.35	0.2040	EO-PE vs. control 0.7679 LO-PE vs. control 0.1812 LO-PE vs. EO-PE 0.5412
Height (cm), mean ± SD	1.68 ± 0.06	1.64 ± 0.05	1.65 ± 0.06	0.0515	EO-PE vs. control 0.0453 LO-PE vs. control 0.2247 LO-PE vs. EO-PE 0.6861
Weight before pregnancy (kg), mean ± SD	63.40 ± 10.45	67.96 ± 13.48	69.30 ± 13.41	0.2190	EO-PE vs. control 0.4152 LO-PE vs. control 0.2141 LO-PE vs. EO-PE 0.9234
Weight at the end of pregnancy (kg), mean ± SD	77.04 ± 11.28	80.96 ± 15.25	85.26 ± 16.25	0.1300	EO-PE vs. control 0.6119 LO-PE vs. control 0.1083 LO-PE vs. EO-PE 0.5419
Weight difference (kg), mean ± SD	13.64 ± 3.25	13.00 ± 4.54	15.96 ± 6.42	0.0847	EO-PE vs. control 0.8942 LO-PE vs. control 0.2178 LO-PE vs. EO-PE 0.0915
BMI before pregnancy (kg/m^2^), mean ± SD	22.41 ± 3.32	25.27 ± 4.74	25.19 ± 4.19	0.0243	EO-PE vs. control 0.0462 LO-PE vs. control 0.0460 LO-PE vs. EO-PE 0.9973
BMI before pregnancy, n (%)					
<18.5, underweight	2 (8.0%)	0 (0.0%)	2 (7.4%)	0.0134 ^#^	
18.5–24.9, normal	21 (84.0%)	12 (50.0%)	13 (48.2%)		
25.0–29.9, overweight	1 (4.0%)	8 (33.3%)	9 (33.3%)		
>30.0, obese	1 (4.0%)	4 (16.7%)	3 (11.1%)		
BMI at the end of pregnancy (kg/m^2^), mean ± SD	27.24 ± 3.49	30.10 ± 5.42	31.08 ± 4.80	0.0113	EO-PE vs. control 0.0837 LO-PE vs. control 0.0104 LO-PE vs. EO-PE 0.7327
BMI difference (kg/m^2^), mean ± SD	4.83 ± 1.10	4.84 ± 1.72	5.90 ± 2.21	0.0461	EO-PE vs. control 0.9995 LO-PE vs. control 0.0775 LO-PE vs. EO-PE 0.0869
Systolic blood pressure (mmHg), mean ± SD	125.56 ± 7.48	174.91 ± 7.65	168.96 ± 12.20	<0.0001	EO-PE vs. control <0.0001 LO-PE vs. control <0.0001 LO-PE vs. EO-PE 0.0767
Diastolic blood pressure (mmHg), mean ± SD	80.48 ± 4.81	106.61 ± 6.20	103.96 ± 8.14	<0.0001	EO-PE vs. control <0.0001 LO-PE vs. control <0.0001 LO-PE vs. EO-PE 0.3382
Daily urinary protein loss, mean ± SD	—	3.41 ± 4.48	1.69 ± 2.53	0.1233 ^^^	
Total protein in urine, mean ± SD	—	5.62 ± 0.38	5.69 ± 0.44	0.6664 ^^^	
Week of hypertension onset, mean ± SD	—	28.21 ± 3.41	32.37 ± 2.39	<0.0001 ^^^	
Headache, n (%)					
Yes	0 (0.0%)	12 (48.0%)	4 (15.4%)	0.0002 ^#^	
No	25 (100.0%)	13 (52.0%)	22 (84.6%)		
Number of past pregnancies, n (%)					
Primiparas	15 (60.0%)	17 (70.8%)	22 (81.5%)	0.2228 ^#^	
Multiple births (2 to 6 pregnancies)	10 (40.0%)	7 (29.2%)	5 (18.5%)		
Method of pregnancy termination, n (%)					
Cesarean section	10 (40.0%)	24 (100.0%)	25 (92.6%)	<0.0001 ^#^	
Spontaneous labor	10 (40.0%)	0 (0.0%)	0 (0.0%)		
Vacuum extraction	5 (20.0%)	0 (0.0%)	2 (7.4%)		

mean ± SD; *p*—ANOVA; * *p*—Tukey’s HSD; ^#^ Fisher’s exact test; ^^^ Two-Sample *t*-test.

### 4.2. Samples

In order to conduct this study, venous blood and morning urine samples were obtained from both groups and pipetted into cryopure test tubes intended for the refrigeration of biological samples.

Blood sera and urine were stored at −80 °C.

### 4.3. Methodology

#### 4.3.1. Measurement of Protein Concentrations

The patients that were included in this study were analyzed based on the concentrations of six proteins which are included in the Bio-Plex Pro RBM Human Kidney Toxicity Panel 1, Bio-Rad, Hercules, CA, USA: calbindin, clusterin, GSTP1, IL-18, KIM-1, and MCP-1.

The measurements in this study were conducted according to the instruction leaflet provided by the manufacturer.

The kidney toxicity protein profile was determined using the following methods: magnetic separation and flow cytometry. The kit contains the reagents, including standards and quality controls, and a reaction site: a 96-well plate. The Bio-Plex method is based on the use of primary antibodies conjugated with fluorescent magnetic beads, which have different colors and are directed against targeted markers [35]. Concisely, serum and urine samples, standards, and quality controls were added to separate wells containing the antibody-coupled beads, and then, the mixture was incubated for an hour at room temperature. After the incubation period, the washing step was conducted 3 times to remove the non-bound protein molecules. Afterwards, detection antibody–biotin reporters were added to the beads and made a sandwich-like structure by binding with the biomarkers. The next step included the addition of a streptavidin–phycoerythrin conjugate, which led to the establishment of the final complex. In this complex, streptavidin attaches to the antibody–biotin reporters, and phycoerythrin works as a fluorescent tag/marker.

The concentrations of calbindin, clusterin, GSTP1, IL-18, KIM-1, and MCP-1 were measured by the flow cytometer Bio-Plex 200, Bio-Rad, Hercules, CA, USA. It is equipped with two diodes, one emitting red light with a wavelength of 635 nm and the second one emitting green light with a wavelength of 532 nm.

Data acquisition was performed using Bio-Plex Manager software version 6.2, Bio-Rad, Hercules, CA, USA. The calibration and verification of the software were performed before the analysis. The standard curve was created using manufacturer-supplied standards for each of the proteins. Quality control was carried out in order to verify the correctness of the analysis.

#### 4.3.2. Statistical Analyses

All statistical analyses were performed using R software version 4.1.2 R Development Core Team, Wienna, Austria (accesed on 5 January 2022). The normality of distribution was assessed using the Shapiro–Wilk test. Variables with a normal distribution are presented as the mean ± standard deviation (SD), while those with a non-normal distribution are shown as the median and interquartile range (IQR).

The homogeneity of variances was evaluated using Levene’s test. The following group comparisons were performed: PE vs. control, EO-PE vs. control, LO-PE vs. control, and EO-PE vs. LO-PE. Differences between groups were assessed using a Student’s *t*-test (for normally distributed data) or the Mann–Whitney U test (for non-normally distributed data). For comparisons of more than two independent groups, one-way ANOVA with Tukey’s HSD post hoc test was used for normally distributed variables, while the Kruskal–Wallis test with Dunn’s post hoc test was applied for non-Gaussian distributions.

Categorical variables are presented as counts and percentages and were compared using Pearson’s χ^2^ test or Fisher’s exact test, depending on the expected frequencies.

Spearman’s rank correlation with Holm correction for multiple comparisons was used to examine relationships between non-normally distributed protein concentrations.

The predictive value of protein levels for preeclampsia risk was evaluated using Receiver Operating Characteristic (ROC) curve analysis, assessing the Area Under the Curve (AUC). Optimal cut-off points were determined based on the highest combined sensitivity and specificity.

The AUC values were interpreted as follows: 0.90–1.00 = excellent, 0.80–0.90 = good, 0.70–0.80 = acceptable, 0.60–0.70 = poor, and <0.60 = failed. The cut-off points were determined using Youden’s index, and the statistical significance of the AUC values was assessed using DeLong’s non-parametric method. For all analyses, statistical significance was set at *p* < 0.05.

## 5. Conclusions

In conclusion, some proteins available in the Bio-Plex Pro RBM Human Renal Toxicity Panel 1, Bio-Rad, Hercules, CA, USA, can be used as molecular markers of kidney injury in PE. Calbindin 1, clusterin, GSTP1, KIM-1, and MCP-1 concentrations in maternal plasma could be used in the monitoring of kidney injury in preeclamptic women; however, there is a need to perform longitudinal studies. The KIM-1 protein may be advantageous as a laboratory marker of LO-PE. The lack of correlation between the clinical features of PE and the protein plasma and urine concentrations indicates the necessity for further studies in this area, in particular longitudinal studies. The present study provides a good framework for future studies to explore new biomarkers that better correlate with the course of PE and renal injury in this group of patients.

It is worth noting that such research would be particularly useful in the early stage of pregnancy in women with a high risk of developing PE throughout the course of pregnancy. Further longitudinal studies of high-risk women could clarify the prognostic value of the biomarkers identified in this pilot study. These biomarkers can be measured before visible clinical onset, which would address an unmet need in PE management.

## Figures and Tables

**Table 1:** The descriptive characteristics of the concentrations of the studied proteins in the study group.

Protein	N	Mean	SD	Median	Min.	Max.	1 Q	3 Q
Serum								
Calbindin (ng/mL)	76	883.53	131.7	892.86	251.62	1113.75	835.7	967.7
Clusterin (ng/mL)	76	116,351.27	47,171.17	113,500.37	18.43	288,133.68	88,137.83	149,596.61
GSTP1 (ng/mL)	75	22	21.97	16.72	0.21	149.74	13.21	22.78
IL-18 (ng/mL)	76	2.58	0.49	2.55	0.75	4.74	2.35	2.75
KIM-1 (ng/mL)	76	3.17	0.48	3.14	0.74	4.16	2.93	3.37
MCP-1 (ng/mL)	75	0.98	0.22	0.96	0.37	1.5	0.81	1.12
Urine								
Calbindin (ng/mL)	56	257.67	234.62	187.31	6.32	948.38	81.68	328.56
Clusterin (ng/mL)	73	99.39	192.8	37.26	0.46	1180.83	10.75	88.15
GSTP1 (ng/mL)	70	70.18	134.18	23.72	0.03	951.25	5.89	77.53
IL-18 (ng/mL)	54	0.18	0.24	0.12	0.01	1.61	0.06	0.23
KIM-1 (ng/mL)	73	0.98	1.24	0.58	0.03	6.72	0.28	1.28
MCP-1 (ng/mL)	70	0.92	1.28	0.56	0.05	9.09	0.21	1.19

SD—standard deviation; min.—minimum value; max.—maximum value; Q—quartile.

**Table 2:** Results of determination of serum and urine protein concentrations of patients in study and control groups.

Protein	PE Group (N = 51)	Control Group (N = 25)	*p*
Serum			
Calbindin (ng/mL)	925.12 (849.40–988.90)	838.47 (772.11–892.86)	0.0024
Clusterin (ng/mL)	124,693.50 (97,071.73–154,823.30)	96,323.58 (69,449.51–116,498.70)	0.0018
GSTP1 (ng/mL)	19.20 (15.67–26.03)	13.08 (10.76–15.82)	0.0001
IL-18 (ng/mL)	2.61 (2.35–2.75)	2.41 (2.22–2.75)	0.1942
KIM-1 (ng/mL)	3.25 (3.00–3.51)	3.03 (2.93–3.14)	0.0042
MCP-1 (ng/mL)	0.99 (0.87–1.15)	0.84 (0.77–1.03)	0.0228
Urine			
Calbindin (ng/mL)	155.77 (85.26–291.17)	264.94 (86.69–350.51)	0.4938
Clusterin (ng/mL)	35.70 (14.49–84.69)	39.52 (9.25–96.36)	0.7867
GSTP1 (ng/mL)	24.23 (6.68–76.85)	23.22 (5.32–89.70)	0.7357
IL-18 (ng/mL)	0.12 (0.05–0.23)	0.09 (0.06–0.20)	0.8422
KIM-1 (ng/mL)	0.61 (0.32–1.60)	0.48 (0.16–0.72)	0.0782
MCP-1 (ng/mL)	0.61 (0.26–1.29)	0.45 (0.18–0.71)	0.2098

Median (IQR—interquartile range); *p*—Mann–Whitney U test.

**Table 3:** Results of determination of serum and urine protein concentrations of patients in EO-PE, LO-PE, and control groups.

Protein		EO-PE (N = 24)		LO-PE (N = 27)	Control Group	*p*
Serum						
Calbindin (ng/mL)		919.76 (854.86–967.75)		925.12 (849.40–988.90)	838.47 (772.11–892.86)	0.0095
Clusterin (ng/mL)		123,330.50 (97,852.67–156,932.50)		124,840.30 (97,071.73–151,855.10)	96,323.58 (69,449.51–116,498.70)	0.0071
GSTP1 (ng/mL)		20.15 (16.26–28.51)		18.57 (14.50–22.78)	13.08 (10.76–15.82)	0.0003
IL-18 (ng/mL)		2.48 (2.32–2.65)		2.61 (2.41–2.78)	2.41 (2.22–2.75)	0.1397
KIM-1 (ng/mL)		3.27 (3.11–3.49)		3.25 (2.93–3.49)	3.03 (2.93–3.14)	0.0109
MCP-1 (ng/mL)		0.99 (0.85–1.15)		1.03 (0.87–1.15)	0.84 (0.77–1.03)	0.0706
Urine						
Calbindin (ng/mL)		177.84 (100.18–475.25)		153.53 (78.48–249.23)	264.94 (86.69–350.51)	0.5449
Clusterin (ng/mL)		19.60 (6.86–80.59)		40.64 (23.04–86.31)	39.52 (9.25–96.36)	0.2978
GSTP1 (ng/mL)		11.61 (4.51–98.92)		35.38 (16.19–66.33)	23.22 (5.32–89.70)	0.5409
IL-18 (ng/mL)		0.10 (0.03–0.14)		0.14 (0.07–0.28)	0.09 (0.06–0.20)	0.1929
KIM-1 (ng/mL)		0.37 (0.27–0.90)		0.73 (0.54–1.69)	0.48 (0.16–0.72)	0.0235
MCP-1 (ng/mL)		0.47 (0.20–1.25)		0.69 (0.36–1.29)	0.45 (0.18–0.71)	0.3325
	Serum			Urine		
p*	Control vs. EO-PE	Control vs. LO-PE	EO-PE vs. LO-PE	Control vs. EO-PE	Control vs. LO-PE	EO-PE vs. LO-PE
Calbindin	0.0272	0.0156	0.8023	0.9635	0.9866	0.7831
Clusterin	0.0145	0.0171	0.7888	0.5949	0.6846	0.3768
GSTP1	0.0003	0.0075	0.2596	0.7657	0.8771	0.8757
IL-18	0.7364	0.1894	0.2696	0.4041	0.7191	0.2145
KIM-1	0.0106	0.0713	0.3717	0.6349	0.0341	0.0730
MCP-1	0.1430	0.0933	0.7632	0.5147	0.4184	0.8760

*p*—Kruskal–Wallis rank sum test. p*—Dunn multiple comparison adjusted with the Holm method.

**Table 4:** Results of correlation in serum and urine proteins of patients in PE and control groups.

Serum						
	Calbindin	Clusterin	GSTP1	IL-18	KIM-1	MCP-1
Calbindin	—	Control: rho = 0.87 *p* < 0.001	Control: rho = 0.36 *p* > 0.999	Control: rho = 0.27 *p* > 0.999	Control: rho = 0.41 *p* > 0.999	Control: rho = 0.52 *p* < 0.001
Clusterin	PE rho = 0.79 *p* < 0.001	—	Control: rho = 0.39 *p* > 0.999	Control: rho = 0.18 *p* > 0.999	Control: rho = 0.24 *p* > 0.999	Control: rho = 0.41 *p* > 0.999
GSTP1	PE rho = 0.41 *p* = 0.134	PE rho = 0.49 *p* = 0.013	—	Control: rho = 0.42 *p* > 0.999	Control: rho = 0.24 *p* > 0.999	Control: rho = 0.14 *p* > 0.999
IL-18	PE rho = 0.63 *p* < 0.001	PE rho = 0.43 *p* = 0.101	PE rho = 0.35 *p* = 0.620	—	Control: rho = 0.72 *p* = 0.003	Control: rho = 0.66 *p* = 0.023
KIM-1	PE rho = 0.72 *p* < 0.001	PE rho = 0.58 *p* < 0.001	PE rho = 0.39 *p* = 0.237	PE rho = 0.60 *p* < 0.001	—	Control: rho = 0.68 *p* = 0.014
MCP-1	PE rho = 0.63 *p* < 0.001	PE rho = 0.58 *p* < 0.001	PE rho = 0.24 *p* > 0.999	PE rho = 0.61 *p* < 0.001	PE rho = 0.47 *p* = 0.031	—
Urine						
	Calbindin	Clusterin	GSTP1	IL-18	KIM-1	MCP-1
Calbindin	—	Control: rho = 0.80 *p* = 0.004	Control: rho = 0.71 *p* = 0.052	Control: rho = 0.79 *p* = 0.017	Control: rho = 0.79 *p* = 0.006	Control: rho = 0.67 *p* = 0.131
Clusterin	PE rho = 0.57 *p* = 0.012	—	Control: rho = 0.83 *p* < 0.001	Control: rho = 0.70 *p* = 0.124	Control: rho = 0.89 *p* < 0.001	Control: rho = 0.90 *p* < 0.001
GSTP1	PE rho = 0.29 *p* > 0.999	PE rho = 0.69 *p* < 0.001	—	Control: rho = 0.59 *p* = 0.737	Control: rho = 0.88 *p* < 0.001	Control: rho = 0.76 *p* = 0.002
IL-18	PE rho = 0.66 *p* = 0.001	PE rho = 0.62 *p* = 0.002	PE rho = 0.31 *p* > 0.999	—	Control: rho = 0.65 *p* = 0.324	Control: rho = 0.65 *p* = 0.325
KIM-1	PE rho = 0.67 *p* < 0.001	PE rho = 0.81 *p* < 0.001	PE rho = 0.48 *p* = 0.036	PE rho = 0.68 *p* < 0.001	—	Control: rho = 0.81 *p* < 0.001
MCP-1	PE rho = 0.68 *p* < 0.001	PE rho = 0.75 *p* < 0.001	PE rho = 0.47 *p* = 0.060	PE rho = 0.67 *p* < 0.001	PE rho = 0.76 *p* < 0.001	—

**Table 5:** Results of correlation in serum and urine proteins of patients in EO-PE and LO-PE groups.

Serum						
	Calbindin	Clusterin	GSTP1	IL-18	KIM-1	MCP-1
Calbindin	—	LO-PE: rho = 0.68 *p* = 0.008	LO-PE: rho = 0.29 *p* > 0.999	LO-PE: rho = 0.63 *p* = 0.028	LO-PE: rho = 0.72 *p* = 0.002	LO-PE: rho = 0.69 *p* = 0.005
Clusterin	EO-PE rho = 0.92 *p* < 0.001	—	LO-PE: rho = 0.41 *p* > 0.999	LO-PE: rho = 0.23 *p* > 0.999	LO-PE: rho = 0.46 *p* = 0.960	LO-PE: rho = 0.49 *p* = 0.608
GSTP1	EO-PE rho = 0.62 *p* = 0.070	EO-PE rho = 0.62 *p* = 0.071	—	LO-PE: rho = 0.28 *p* > 0.999	LO-PE: rho = 0.32 *p* > 0.999	LO-PE: rho = 0.19 *p* > 0.999
IL-18	EO-PE rho = 0.55 *p* = 0.332	EO-PE rho = 0.60 *p* = 0.114	EO-PE rho = 0.50 *p* = 0.655	—	LO-PE: rho = 0.59 *p* = 0.086	LO-PE: rho = 0.56 *p* = 0.171
KIM-1	EO-PE rho = 0.78 *p* < 0.001	EO-PE rho = 0.74 *p* = 0.002	EO-PE rho = 0.45 *p* = 0.352	EO-PE rho = 0.68 *p* = 0.018	—	LO-PE: rho = 0.30 *p* > 0.999
MCP-1	EO-PE rho = 0.54 *p* = 0.350	EO-PE rho = 0.62 *p* = 0.085	EO-PE rho = 0.33 *p* > 0.999	EO-PE rho = 0.65 *p* = 0.035	EO-PE rho = 0.61 *p* = 0.104	—
Urine						
	Calbindin	Clusterin	GSTP1	IL-18	KIM-1	MCP-1
Calbindin	—	LO-PE: rho = 0.35 *p* > 0.999	LO-PE: rho = 0.18 *p* > 0.999	LO-PE: rho = 0.68 *p* = 0.026	LO-PE: rho = 0.68 *p* = 0.017	LO-PE: rho = 0.56 *p* = 0.318
Clusterin	EO-PE rho = 0.91 *p* < 0.001	—	LO-PE: rho = 0.54 *p* = 0.242	LO-PE: rho = 0.35 *p* > 0.999	LO-PE: rho = 0.72 *p* = 0.002	LO-PE: rho = 0.64 *p* = 0.023
GSTP1	EO-PE rho = 0.46 *p* > 0.999	EO-PE rho = 0.79 *p* = 0.003	—	LO-PE: rho = 0.05 *p* > 0.999	LO-PE: rho = 0.37 *p* > 0.999	LO-PE: rho = 0.38 *p* > 0.999
IL-18	EO-PE rho = 0.72 *p* = 0.707	EO-PE rho = 0.87 *p* = 0.001	EO-PE rho = 0.68 *p* = 0.401	—	LO-PE: rho = 0.57 *p* = 0.323	LO-PE: rho = 0.48 *p* > 0.999
KIM-1	EO-PE rho = 0.70 *p* = 0.332	EO-PE rho = 0.84 *p* < 0.001	EO-PE rho = 0.53 *p* = 0.859	EO-PE rho = 0.80 *p* = 0.021	—	LO-PE: rho = 0.68 *p* = 0.008
MCP-1	EO-PE rho = 0.89 *p* = 0.002	EO-PE rho = 0.81 *p* < 0.001	EO-PE rho = 0.43 *p* > 0.999	EO-PE rho = 0.87 *p* = 0.002	EO-PE rho = 0.84 *p* < 0.001	—

**Table 6:** Descriptive statistics of ROC curves for PE and control groups.

Protein	AUC (95%PU)	SE	Cut-Off Point	Sensitivity	Specificity	*p*
Serum						
Calbindin	0.715 (0.588–0.843)	0.065	909.017	0.840	0.549	0.0009
Clusterin	0.722 (0.600–0.845)	0.063	121,362.837	0.880	0.549	0.0004
GSTP1	0.779 (0.667–0.892)	0.057	15.666	0.750	0.745	0.000001
IL-18	0.592 (0.447–0.737)	0.074	2.447	0.520	0.706	0.2128
KIM-1	0.703 (0.585–0.821)	0.060	3.218	0.800	0.569	0.0007
MCP-1	0.664 (0.523–0.805)	0.072	0.853	0.542	0.804	0.0225
Urine						
Calbindin	0.558 (0.390–0.726)	0.086	243.574	0.556	0.737	0.5010
Clusterin	0.480 (0.332–0.627)	0.075	1.955	1.000	0.078	0.7855
GSTP1	0.525 (0.376–0.675)	0.076	34.525	0.696	0.468	0.7391
IL-18	0.518 (0.350–0.686)	0.086	0.091	0.500	0.632	0.8327
KIM-1	0.631 (0.495–0.767)	0.069	0.187	0.364	0.882	0.0593
MCP-1	0.595 (0.450–0.740)	0.074	0.731	0.773	0.417	0.1998

## Data Availability

The original contributions presented in this study are included in the article. Further inquiries can be directed to the corresponding author.
